# Mesenteric excision and Kono‐S anastomosis trial (MEErKAT): A study protocol for a multicentre, 2 × 2 factorial, randomised controlled, open‐label superiority trial

**DOI:** 10.1111/codi.70212

**Published:** 2025-09-08

**Authors:** Deepak Selvakumar, Jamie Hall, Olivia Hawksworth, Daniel Hind, Stephen Walters, Ines Rombach, Esther Herbert, William Waterworth, Lucy Sibbald, Mark Travis, Daniel Brice, Alan Lobo, Laura Hancock, Steven Brown

**Affiliations:** ^1^ Department of Surgery Manchester University Hospitals NHS Foundation Trust Manchester UK; ^2^ Lydia Becker Institute of Immunology and Inflammation University of Manchester Manchester UK; ^3^ Manchester Academic Health Science Centre University of Manchester Manchester UK; ^4^ Sheffield Centre for Health and Related Research University of Sheffield Sheffield UK; ^5^ Patient and Public Involvement Manchester UK; ^6^ Department of Gastroenterology and Hepatology, Royal Hallamshire Hospital Sheffield Teaching Hospitals NHS Foundation Trust Sheffield UK; ^7^ Department of Surgery Sheffield Teaching Hospitals Sheffield UK

**Keywords:** Crohn's disease, inflammatory bowel disease, surgery

## Abstract

**Aim:**

Controversy exists over whether surgical technique can reduce recurrence following Crohn's resection. This study compares the rate of endoscopic recurrence after different approaches to mesenteric excision (extended/close) and anastomosis (Kono‐S/standard of care) in adult patients undergoing ileocolic resection for primary or recurrent Crohn's disease.

**Method:**

MEErKAT is a UK multicentre, 2 × 2 factorial, randomised, controlled, open‐label superiority trial where participants (target sample size = 308) are blinded and centrally randomised (1:1:1:1) to one of four groups: (1) Kono‐S + extended mesenteric resection. (2) Kono‐S + close mesenteric resection. (3) Standard anastomosis + extended mesenteric resection. (4) Standard anastomosis + close mesenteric resection. The primary outcome is time to endoscopic recurrence of disease (up to 3 years follow‐up). Secondary outcomes include rates of severe and symptomatic recurrence, complications, and quality of life scores. The locality of recurrence will be investigated using endoscopic assessment of the mucosa relative to mucosal tattoos placed at the time of operation. The degree and anastomotic locality of different immune cells will be compared before and after each intervention to better understand the mechanistic processes driving disease recurrence.

**Conclusion:**

This study will robustly evaluate the efficacy of the Kono‐S anastomosis technique and extended mesenteric excision in reducing endoscopic recurrence rates. The additive effect of these techniques and local tissue immune response will be investigated. This will provide important evidence to guide the optimal surgical technique and improve our understanding of the processes leading to recurrent disease.

**What does this paper add to the literature?**

The proposed study will evaluate the efficacy of the Kono‐S anastomosis and extended mesenteric excision in reducing endoscopic recurrence rates for patients undergoing ileocolic resection for primary or recurrent Crohn's disease.

## INTRODUCTION

Crohn's disease (CD) is a relatively common chronically relapsing inflammatory condition of the gastrointestinal tract [1]. Despite advances in medical therapy, a large proportion of patients eventually require resection of diseased bowel, with over one third of these people requiring further surgery within 10 years [2].

Animal models and pathological specimens indicate that patterns of recurrence begin at the mesenteric border of the anastomosis [3, 4, 5]. The mechanisms believed to drive recurrence involve faecal stasis and alterations in the gut microbiome [6]. Some highlight the significance of mesenteric vascular anatomy, emphasising that the mesenteric border of the bowel relies on end arteries, while the antimesenteric border has collateral supply. Therefore, disease in the mesentery is likely to disrupt the blood supply to the mesenteric border before the antimesenteric border, resulting in the observed pattern of ischaemic ulceration [7, 8]. Supporting this theory, studies on strictureplasty show a very low site‐specific surgical recurrence rate, despite leaving diseased bowel and mesentery in place [9].

Alternative theories propose the mesentery as the disease focus, observing elevated visceral fat content, increased lymphatic vascular density at the resection margin, and the presence of granulomata in the mesenteric lymph nodes, all associated with recurrence [10, 11, 12]. This leads to the concept that extended resection is required to eliminate the disease focus.

These theories have prompted surgeons to consider whether the techniques of resection and anastomosis can influence recurrence rates. Different anastomotic configurations and techniques have been tested, yielding inconsistent results [13, 14]. Present consensus supports a wide lumen configuration, achieved through a stapled side‐to‐side technique [15, 16].

Two techniques, despite limited evidence, have gained attention due to a seemingly spectacular reduction in recurrence [17]. The Kono‐S anastomosis has a wide‐lumen, antimesenteric configuration to address the predisposition for mesenteric border recurrence. A systematic review showed several low‐quality studies and one high‐quality randomised controlled trial (RCT) affirming the safety of this technique [17]. The findings also suggested a remarkable 65% reduction in endoscopic recurrence after 6 months (22.2% in the Kono‐S group compared to 62.8%). If this level of reduction in recurrence rates holds true, the Kono‐S technique will have profound implications for disease management. However, there is a need for high‐quality data to better determine the effectiveness of this technique, with some comparative studies ongoing (e.g. NCT 03256240).

An alternative concept proposes the mesentery as the primary driver of disease, advocating for extended resection of the diseased mesentery, with the anastomosis being considered irrelevant to recurrence [18, 19]. The recent evidence supporting extended resection of the mesentery is limited and contradictory. The SPICY randomised controlled trial comparing extended mesenteric resection with conventional mesenteric resection did not show a difference in endoscopic recurrence rates 6 months after surgery (42% in the extended mesenteric group compared to 43% in the mesenteric sparing resection group) [20]. In contrast, the interim results of the MESOCOLIC trial favour a more radical resection in reducing endoscopic recurrence [21]. The authors speculate that the different methods of extended mesenteric excision (high ligation vs. preservation of the ileocolic vessels) are responsible for the observed difference.

There are commonalities with the techniques of Kono‐S anastomosis and extended mesenteric resection that may explain their potential effectiveness. Both isolate the anastomosis from the diseased mesentery. Kono‐S achieves this through a totally antimesenteric anastomosis placed as far away as possible from the mesentery, while extended mesenteric resection removes the theoretical disease driver. A combined approach is technically feasible and may improve efficacy. If either or both interventions result in reduced recurrence, understanding the underlying mechanism of action becomes a crucial question. To explore this, we will examine the locality of any mucosal recurrence. The prevailing notion is that CD arises from the interplay of genetically inheritable traits and environmental factors, including the microbiota, leading to innate and adaptive immune cell‐mediated inflammation [22]. Analysing the immune cell phenotypes in the mucosa of the different combinations of resection and anastomosis will provide insights into the impact each intervention has on the mechanism of inflammation [23, 24, 25]. Examining visceral fat area, anastomotic locality of immune cell populations, with a specific focus on T cell activation and exhaustion, will enable us to explore potential underlying mechanisms of action.

We propose a protocol for a UK multicentre, superiority, 2 × 2 factorial, randomised, open‐label trial with a 1‐year follow‐up (−6 months/+3 months). Participants will be randomised (1:1:1:1) to one of four groups:

1. Kono‐S + extended mesenteric resection;
2. Kono‐S + close mesenteric resection;
3. Standard anastomosis + extended mesenteric resection;
4. Standard anastomosis + close mesenteric resection.

Our trial will investigate relevant clinical and mechanistic outcomes on: (1) the Kono‐S anastomosis; (2) extended mesenteric excision; (3) the locality of recurrence after surgery; and (4) local tissue immune response and its association with surgical recurrence.

The main aim of the study is to compare recurrence after standard mesenteric excision or extended excision and standard anastomosis or Kono‐S anastomosis (with or without extended mesenteric excision).

## METHODS

This protocol has been written according to SPIRIT guidelines.

### Study setting

The study will recruit patients aged over 18 years undergoing ileocolic resection for primary/recurrent CD where an anastomosis is carried out. The study will be run nationally within the United Kingdom across up to 27 centres, recruiting an average of one participant per 4 months over a maximum of 45 months.

### Eligibility criteria

#### Inclusion criteria

- Patients aged 18 years and over.
- Patients undergoing ileocaecal resection for primary/recurrent CD where an anastomosis is carried out.

#### Exclusion criteria

- Patients with markedly extensive inflammation affecting the vascular root of the mesentery seen on imaging or at operation
- Patients undergoing stoma formation proximal to the anastomosis
- Patients who have a contraindication to subsequent colonoscopy
- Patients unable to give full informed consent
- Patients who are pregnant
- Patients who, in the opinion of the principal investigator, do not meet the criteria for relevant surgery

In a very small subset of patients, it may be the case that extensive mesenteric inflammation is only seen intraoperatively, meaning the patient is ineligible for the trial. Participants should therefore not undergo randomisation until the diseased area can be visually assessed intra‐operatively.

### Interventions

There are two groups of mesenteric excisions and two groups of anastomoses.

### Mesenteric excision

#### Extended mesenteric excision

The mesentery is resected up to the origin of the ileocolic trunk but preserving the ileocolic vessels as described, in detail, in the SPICY trial [20, 26]. In participants who have markedly extensive inflammation affecting the vascular root of the mesentery seen on imaging or at operation, these should not undergo extended resection due to the risk of vascular injury and should be excluded from the trial.

#### Close mesenteric excision

The mesentery is resected within 3 cm of the border of the bowel, leaving most of the mesentery in situ.

### Anastomosis

#### Kono‐S

The resected bowel is stapled perpendicular to the mesentery and the stapled ends sutured together to form the supporting column. Seven centimetre antimesenteric enterotomies are made from 1 to 1.5 cm from the stapled resection margin and a side‐to‐side anastomosis created by suturing the enterotomies together.

#### Standard of care

Standard care is essentially surgeons' preference of anastomosis. Anastomosis may utilise staples or sutures and has a configuration of either end to end, functional end to end, or end to side.

The mode of access (open/laparoscopic/robotic), closure technique and post‐operative care are according to usual practice for that participating centre.

For all groups the mesenteric incision will be made proximal to the mesenteric transition zone [27], while the distal incision will be placed where both the mesentery and intestine are macroscopically normal immediately distal to the region of disease. Each technique consists of components familiar to bowel surgeons.

Every participating surgeon will have been mentored for the Kono‐S anastomosis and will have carried out at least 2 procedures outside the trial. A video of each technique will be created and distributed to all surgeons. We will run dedicated training sessions for all surgeons involved in the trial. Two independent reviewers will review images of the resection specimen to ensure adequate quality and extent of mesenteric excision.

Post‐operative follow‐up and colonoscopic assessment at 6–12 months are part of standard practice [28]. Localisation of recurrence will be aided by a tattoo of the mesenteric border of the anastomosis at the time of surgery using carbon black.

### Outcomes

#### Primary outcome

Time to endoscopic recurrence of disease (up to 3 years follow‐up) from the date of randomisation using the Modified Rutgeerts score (≥i2) [29]. With a subgroup analysis of i2a and i2b groups.

Endoscopic recurrence is an early surrogate for surgical recurrence and is frequently used as a primary outcome in related previous trials [21, 30] and ongoing trials. For quality assurance, the endoscopist will be blinded, and the Modified Rutgeerts score will be checked by two independent and blinded assessors.

#### Secondary outcomes

- Incidence of endoscopic recurrence with a Modified Rutgeerts score ≥i2b at 3 years follow‐up.
- Incidence of severe endoscopic recurrence (Modified Rutgeerts score ≥i3) at 3 years follow‐up.
- Clinician and patient‐reported symptomatic recurrence up to 3 years [31].
- Quality of life (EQ‐5D‐5L) [32] assessed at baseline, 6 weeks and 12 months post‐surgery.
- Surgical recurrence up to 3 years (clinician and patient reported).
- Radiological and surgical anastomotic leak as defined by the latest consensus [33]; other complications for each intervention assessed at the time of surgery, 6 weeks and 12 months post‐intervention.

#### Mechanistic outcomes

The degree and anastomotic locality of different immune cell populations, especially CD8+ T cells, will be compared before and after each intervention utilising high‐parameter flow cytometry (Appendix 3). This will be studied using matched mucosal samples taken at the time of surgery and at endoscopic follow‐up, in a minimum of 140 participants.

### Participant timeline

**FIGURE 1:**
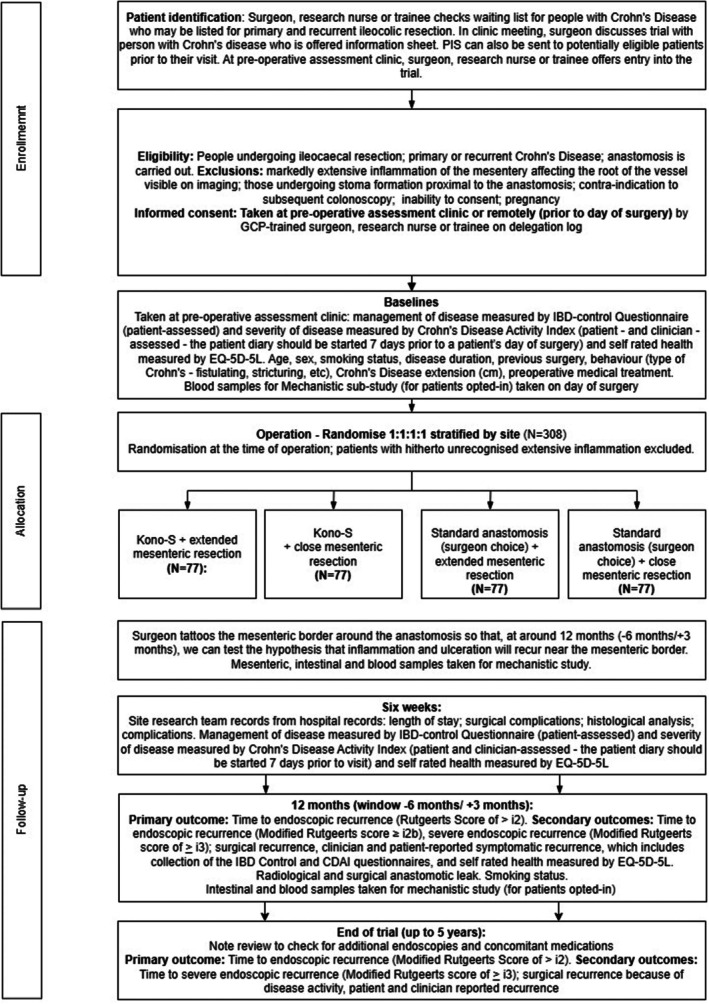
Study flowchart demonstrating participant journey through the trial from identification, enrolment, treatment allocation and follow‐up.

A study flowchart (Figure 1) and the assessments schedule (Table 1) demonstrate the participant journey through the study.

**TABLE 1:** Schedule of assessments during the study.

	Baseline	Operation	6 weeks	12 months (−6 to +3 months)	Study end (up to 5 years)
Eligibility assessment	X	X			
Consent	X				
Medical history	X				
Concomitant medications	X		X	X	X
Demographics	X				
IBD‐Control	X		X	X	
CDAI	X		X	X	
EQ‐5D	X		X	X	
Randomisation		X			
Mesenteric disease activity index		X			
Adverse events		X	X	X	
Colonoscopy				X	X (via note review)
Blood samples	X			X	
Mucosal/mesenteric biopsies		X		X	
Surgical recurrence				X	X (via note review)

### Sample size

The primary outcome will be the time to endoscopic recurrence (ER) post‐randomisation (Modified Rutgeerts score ≥ i2). All participants will be followed up for a minimum of 6 months post‐randomisation and up to a maximum of 3 years. The best existing data indicates ER rates of approximately 65% on conventional surgery and 24% on Kono‐S surgery at 12 months [34]. Other published data on the rate of endoscopic recurrence after conventional surgery varies from 58% to 93% [28, 35, 36, 37]. The systematic review unfortunately found no published data on the ER rates after close or extended mesenteric resection [16]. In a survey of 34 surgeons, 71% were persuaded to change practice based on a reduction in endoscopic recurrence to 30% or less after 12 months.

The sample size calculation for the 2 × 2 factorial design assumes: 90% power; 5% (two‐sided) significance level; and estimated reduction in 1‐year endoscopic recurrence rates from 52.5% to 32.5%. Using the Freedman method a total of 112 recurrences are required [38]. After accounting for surgeon effects (assuming each of 12 sites would have 2 surgeons, an ICC of 0.01 and 15 patients per surgeon) and an attrition rate of 3%, the target sample size is 154 per group for each comparison.

### Recruitment

Potential participants will be discussed at MDT meetings and identified at the time of pre‐operative assessment before the day of surgery. Potential participants will be approached either at their clinic visit prior to surgery or pre‐operative assessment. The study will be run nationally within the United Kingdom across up to 27 centres, recruiting one participant per month over a maximum of 45 months.

### Allocation

Once consent has been obtained (Appendix 1), baseline data recorded and eligibility confirmed, participants will be centrally randomised using the CTRU online randomisation system (SCRAM). Randomisation will occur intraoperatively when all eligibility criteria are met. The doctor or research nurse will access the web‐based randomisation system, enter patient demographic details (ID, date of birth) and the treatment allocation will be returned.

Participants will be allocated using a computer‐generated pseudo‐random list, stratified by centre, with random permuted blocks of varying sizes. The sequence will be restricted by authorisation until analyses are complete.

### Blinding

As there is no difference between the interventions in abdominal access or closure, it is easy to blind the participant. Those assessing the 12‐month endoscopic outcomes will be blinded to the allocation. Endoscopists may recognise the Kono‐S anastomosis in the bowel configuration but will not be directly involved in the study. The degree of mesenteric excision will not be apparent during colonoscopy.

### Data collection

Baseline data will be collected by a research nurse or clinician using specific case report forms (Table 1). Participants will be given a diary to complete 7 days prior to the day of surgery to enable the Crohn's Disease Activity Index (CDAI) to be calculated (Appendix 2). Operative details will be recorded by the operating surgeon. Six‐week and 6‐ to 12‐month follow‐up data will be collected by a research team member who is blind to allocation. Standard colonoscopic follow‐up will be collected by a colonoscopist not involved in the trial. Colonoscopists will collect mucosal biopsies and recurrence data, relative to the small bowel mesenteric tattoo. At the end of the study (12–57 months after surgery) further data will be collected by a team member who is blind to allocation.

Participants may withdraw their consent for the study at any time. Although the participant is not required to give a reason for discontinuing their study treatment, a reasonable effort will be made to establish this reason while fully respecting the participants’ rights.

### Data management

Participant confidentiality will be respected, and the principles of the UK Data Protection Act (DPA) followed. The investigator will ensure that identifiable data is kept securely.

All participants will be assigned a unique study ID number at screening that will link the clinical information collected for them on the study database. All CRFs will only identify the participant by their study ID number.

Study records, including source data, will be stored for 10 years after the completion of the study by participating sites, before being destroyed. Access will be restricted to authorised individuals.

Data management will be provided by the University of Sheffield Clinical Trials Research Unit (CTRU) and a separate data management plan (DMP) will detail activities for the study in accordance with local SOPs.

### Statistical methods

The primary outcome is the time to endoscopic recurrence (ER), over a follow‐up of up to 3 years, defined as a Modified Rutgeerts score [≥i2]. Patients without a reported ER will be censored at their last known date of not having had ER. The primary effectiveness analysis, on the intention‐to‐treat (ITT) sample, will compare the time to ER, between the two factors (Kono‐S vs. standard anastomosis surgery; extended mesenteric resection vs. close mesenteric resection) using a mixed‐effects parametric survival model with random effects for centre and surgeon and fixed effects for the two factors. The model will be implemented using a Weibull survival distribution.

It is anticipated that there will be no interaction between the two factors. To test this assumption, the initial statistical model will include an interaction (Kono‐S (yes or no) vs. extended mesenteric resection (yes or no)) term between the two factors. We will report the estimate of the interaction term and its associated 95% confidence interval (CI).

If the CI for the hazard ratios (HR) for the interaction term shows no evidence of an interaction, then we will analyse the data, without the interaction term, using the simpler factorial design with the two main factors. The CIs for the HRs for the Kono‐S versus standard anastomosis contrast and extended mesenteric resection versus close resection contrasts will be reported from this simpler model. If the CI for the HR for the interaction term demonstrates evidence of an interaction, then we will analyse the data using the four randomised groups separately, with standard anastomosis and close mesenteric resection as the reference treatment. The treatment effects and corresponding 95% CIs will be presented for all relevant comparisons.

We will complement the ITT analysis of the primary outcome with several sensitivity analyses. A per protocol analysis will estimate the efficacy of the Kono‐S versus standard anastomosis and extended versus close mesenteric resection in participants who adhere to the main aspects of the protocol. For participants who do not have a colonoscopy during the trial, or whose Modified Rutgeerts score was not completed, missing data will be imputed through best‐ and worst‐case scenarios to investigate the impact of assuming informative missingness (missing not at random assumptions).

Secondary endpoints will be analysed as follows: Time‐to‐event outcomes will be analysed as per the primary outcome. Binary outcomes will be compared between the two factors ((1) Kono‐S vs. standard anastomosis and (2) extended vs. close mesenteric resection) using a multi‐level mixed effects logistic regression model with adjustment for baseline covariates, with associated ORs and 95% CIs. Absolute risk differences with 95% CIs will also be presented for binary outcomes. Continuous outcomes will be analysed using a multi‐level mixed effects regression model with adjustment for baseline covariates. All multi‐level models will use the same covariates as the primary analysis model. The serious adverse event (SAE) rates in the post‐randomisation period will be compared between the four randomised groups using a chi‐squared test and 95% CIs for each of the four randomised groups. We will also count the total number of SAEs experienced by each patient and compare counts using a Poisson generalised linear model (GLM) and reporting the risk ratio and associated 95% CIs.

Regardless of the statistical significance of the overall effect, exploratory subgroup analyses will be carried out for the primary outcome (ER). We will carry out subgroup analyses to examine if treatment effects differ based on patient demographics, disease phenotype and medical treatment history. As this trial is not formally powered for subgroup analyses, all subgroup effects will be considered exploratory, and *p*‐values will not be presented.

### Data monitoring

The data monitoring and ethics committee (DMEC) will consist of an independent statistician and at least two independent physicians with research experience. The DMEC will review reports provided by the CTRU to assess the progress of the study, the safety data and the critical endpoint data as required. The DMEC will meet every 6 months. There will be no interim analyses (other than for the purposes of the blinded internal pilot) or definitive stopping guidelines, but the DMEC may request unblinded data or study termination on grounds of safety/futility.

### Harms

All Adverse Events (AEs) will be recorded on the adverse event report form, within the participant CRF. Sites are asked to enter all available information onto the study database as soon as possible after the site becomes aware of the event.

Once an SAE has been identified, a member of the site research team will complete an SAE form, notify the site's PI and send this to the CTRU.

SAEs which are related and unexpected will be reported to the sponsor and we will expedite these to the Research Ethics Committee (REC) within 15 days of becoming aware. The DMEC and TSC will also receive information on all AEs and SAEs.

### Auditing

Central and/or on‐site monitoring will be undertaken at a level appropriate to the detailed risk assessment. The level of risk will be agreed with the Sponsor and will be documented in the Trial Monitoring Plan (TMP).

Regular on‐site monitoring visits will occur throughout the study where the Monitor will verify that the:

- Data are authentic, accurate and complete.
- Safety and rights of the patient are being protected.
- Study is conducted in accordance with the approved protocol and study agreements, GCP and all applicable regulatory requirements.

A central review of consent forms will also be completed, and sites will be requested to post consent forms to CTRU on an ongoing basis.

### Ethics and dissemination plan

This study has been granted all necessary ethical approvals from the National research ethics committee (REC: 22/NE/0041). Any protocol amendments will be submitted and approved by the HRA and REC committee. Sheffield CTRU will communicate amendments approved by the funder and HRA to all relevant parties.

All clinicians responsible for recruiting patients to the trial will be trained in Good Clinical Practice (GCP). Participant confidentiality will always be respected, and the principles of the UK Data Protection Act (DPA) will be followed.

Results of the study will be disseminated through peer reviewed scientific journals and at clinical and academic conferences, as well as submission of a final report to the funder, which will be made available online.

We aim to change policy and practice, giving patients greater understanding of available options and the trade‐offs involved. Open access publication will ensure findings are widely available. The Association of Coloproctology of Great Britain and Ireland (ACPGBI), which promotes care of patients with bowel disease, will communicate study findings. Lay members of the study group will help write plain language summaries to communicate findings to the public over a range of media platforms.

Our PPI representatives have the capacity to act as ambassadors for the trial and will inform their peers in other PPI forums and the wider public over the course of the trial.

## DISCUSSION

Identifying the optimal treatment strategy, including the safety, efficacy, and timing of surgery in CD is set as a research priority by The James Lind Alliance and the Association of Coloproctology [39, 40]. Reducing disease recurrence rates or the need for adjuvant medical therapy may encourage patients to opt for surgery earlier. From a health economic perspective, early surgical intervention can also provide significant cost savings compared to prolonged medical therapy [41, 42]. Ultimately, a low recurrence rate after surgery may fundamentally change practice, with early surgery becoming the norm rather than the last resort [43].

The Kono‐S anastomosis and extended mesenteric resection are two techniques which demonstrate seemingly spectacular reduction in recurrence rates and could be readily implemented into the current management of ileocolic CD. However, the current evidence base is limited and there is a clear need to evaluate both the individual and additive effects of these techniques on recurrence rates. The SPICY randomised controlled trial did not show that extended mesenteric resection is superior to conventional resection regarding endoscopic recurrence of Crohn's disease, but it did not consider anastomotic technique and had no mechanistic arm to help explain recurrence after surgery for Crohn's disease [20]. In contrast, the interim results of the MESOCOLIC trial favoured a more radical resection in reducing endoscopic recurrence [21]. The authors speculate that the different methods of extended mesenteric excision are responsible for the observed difference. They recommend high ligation of the ileocolic vessels, whereas the SPICY trial preserved these vessels, and this is our approach in the MEErKAT trial. Preservation of the ileocolic vessels prevents a longer segment of colon being excised and any thickened mesentery affected by ileal Crohn's is inferior to the ileocolic vessels.

Our trial will evaluate the efficacy of extended mesenteric excision and Kono‐S anastomosis both individually and in combination but also investigates the locality of recurrence after surgery and characterises the local tissue immune response. This mechanistic arm will improve our understanding of the biological processes driving recurrence after surgery and help to identify which patients may be at high risk of recurrence.

In accordance with international guidelines, we will use ileocolonoscopy to assess for recurrence 6–12 months after surgery, with a Modified Rutgeerts score > i2 defining endoscopic recurrence. Stratification into i2a and i2b subgroups and image review centrally by two independent blinded assessors familiar with the score and the endoscopic appearance of different anastomotic configurations will overcome some of the concerns regarding the reproducibility and interpretation of the score [44, 45]. The combination of efficient trial design and robust quality assurance will help to provide high‐quality evidence to inform surgical practice.

## AUTHOR CONTRIBUTIONS

**Deepak Selvakumar:** Writing – original draft; writing – review and editing; methodology. **Jamie Hall:** Conceptualization; visualization; writing – original draft; methodology; project administration; funding acquisition; writing – review and editing. **Olivia Hawksworth:** Conceptualization; funding acquisition; writing – original draft; writing – review and editing; visualization; methodology; project administration. **Daniel Hind:** Conceptualization; methodology; funding acquisition. **Stephen Walters:** Conceptualization; methodology; funding acquisition; software; formal analysis; writing – original draft; writing – review and editing; validation. **Ines Rombach:** Conceptualization; methodology; data curation; formal analysis; writing – original draft; writing – review and editing. **Esther Herbert:** Conceptualization; methodology; data curation; formal analysis; writing – original draft; writing – review and editing. **William Waterworth:** Conceptualization; funding acquisition. **Lucy Sibbald:** Conceptualization; funding acquisition. **Mark Travis:** Conceptualization; investigation; writing – original draft; writing – review and editing; formal analysis; methodology; validation. **Daniel Brice:** Methodology; data curation; investigation; formal analysis. **Alan Lobo:** Conceptualization; methodology; investigation; funding acquisition; writing – review and editing. **Laura Hancock:** Conceptualization; investigation; methodology; supervision; writing – original draft; writing – review and editing; funding acquisition; formal analysis; resources. **Steven Brown:** Conceptualization; investigation; funding acquisition; writing – original draft; writing – review and editing; methodology; formal analysis; supervision; resources.

## FUNDING INFORMATION

This study is funded by National Institute for Health Research (NIHR) Efficacy and Mechanism Evaluation (EME) Programme, an MRC and NIHR partnership (NIHR 131988). The views expressed in this protocol are those of the authors and not necessarily those of the MRC, NIHR or the Department of Health and Social Care. The funder has reviewed the research protocol but will have no role in data collection, analysis, data interpretation, report writing or in the decision to submit the report for publication. The funder has approved the selection of members for oversight committees.

## CONFLICT OF INTEREST STATEMENT

The authors declare there are no conflicts of interest.

## ETHICS STATEMENT

The trial will be conducted in accordance with the UK Policy Framework for Health and Social Care Research and Sheffield Clinical Trials Research Unit (CTRU) standard operating procedures. This study has been granted all necessary ethical approvals (REC 22/NE/0041).

## PATIENT CONSENT STATEMENT

Patients will provide written informed consent prior to trial participation.

## TRIAL REGISTRATION

The study has been registered with ISRCTN (ISRCTN: 16900055).

## Supporting information


Data S1.

## Data Availability

Data sharing not applicable to this article as no datasets were generated or analysed during the current study.
