# Supplementary material for: Mesenteric excision and Kono‐S anastomosis trial (MEErKAT): A study protocol for a multicentre, 2 × 2 factorial, randomised controlled, open‐label superiority trial
Source: Colorectal Dis. 2025 Sep 8;27(9):e70212. doi: 10.1111/codi.70212 (PMC12417623; doi:10.1111/codi.70212)
Supplement: Supplementary file 1 — Data S1. [file CODI-27-0-s001.docx]

**Appendices**

**
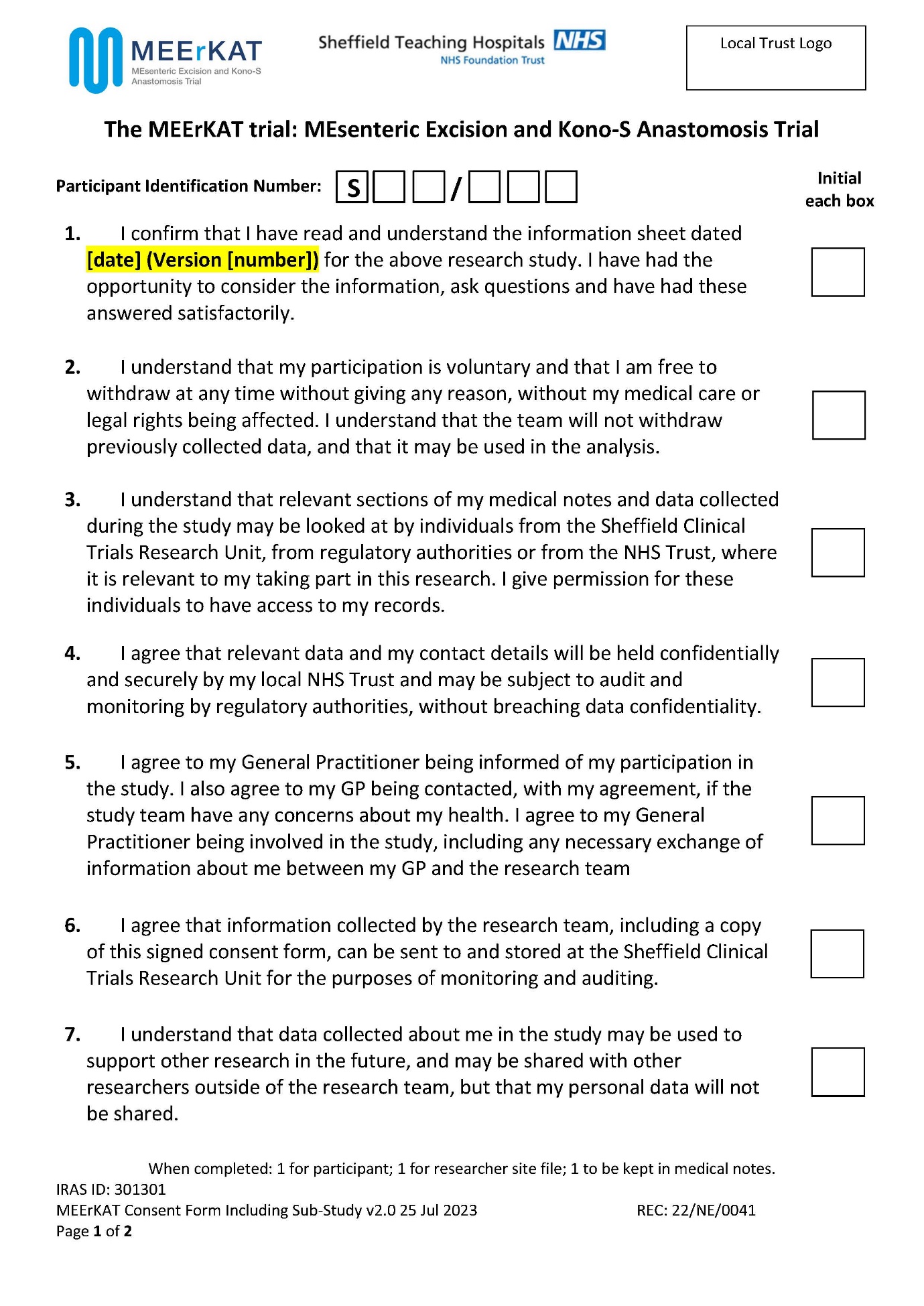
Appendix 1. Model consent form for patients**

**Appendix 1 continued. Model consent form for patients**

**
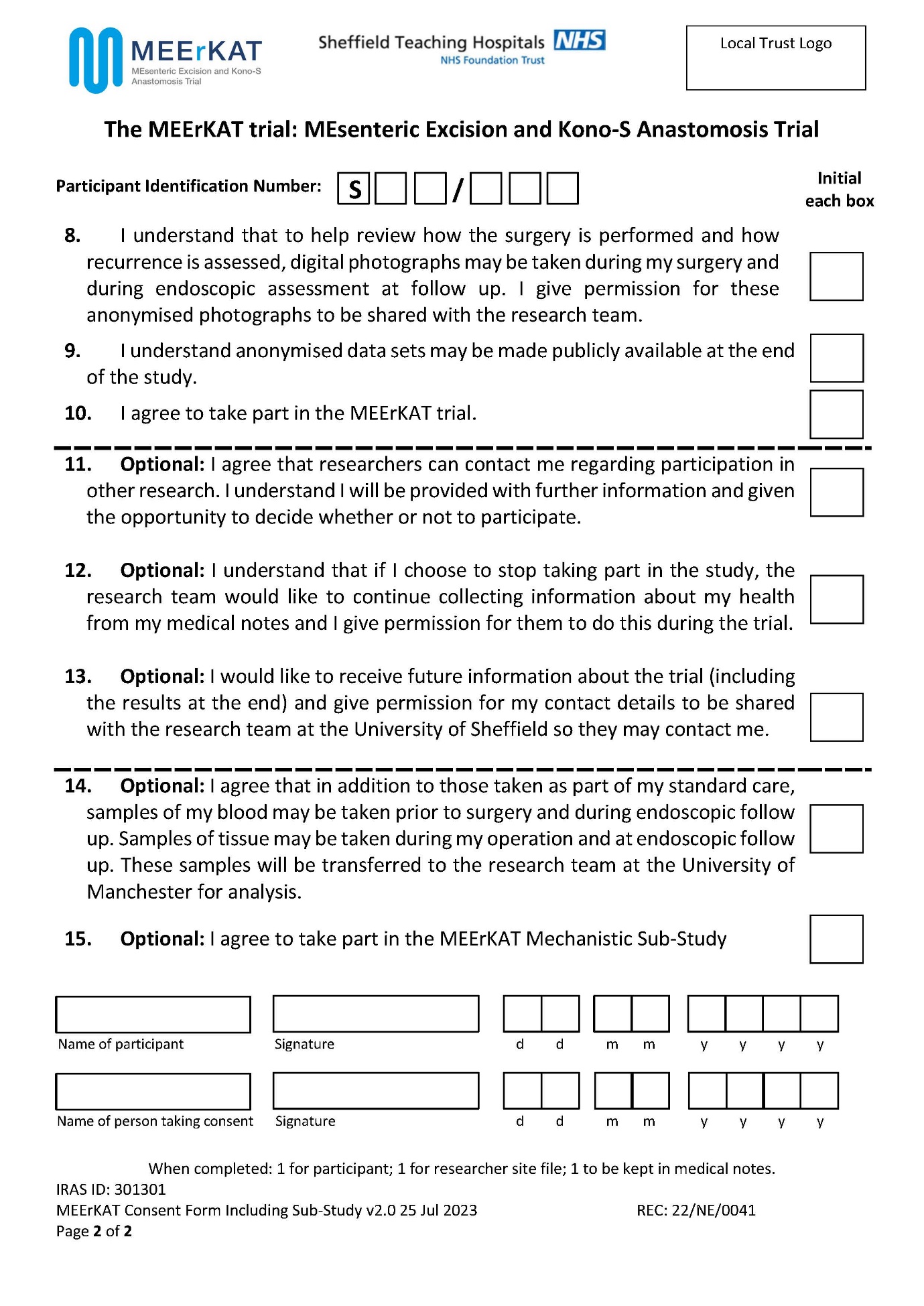
**


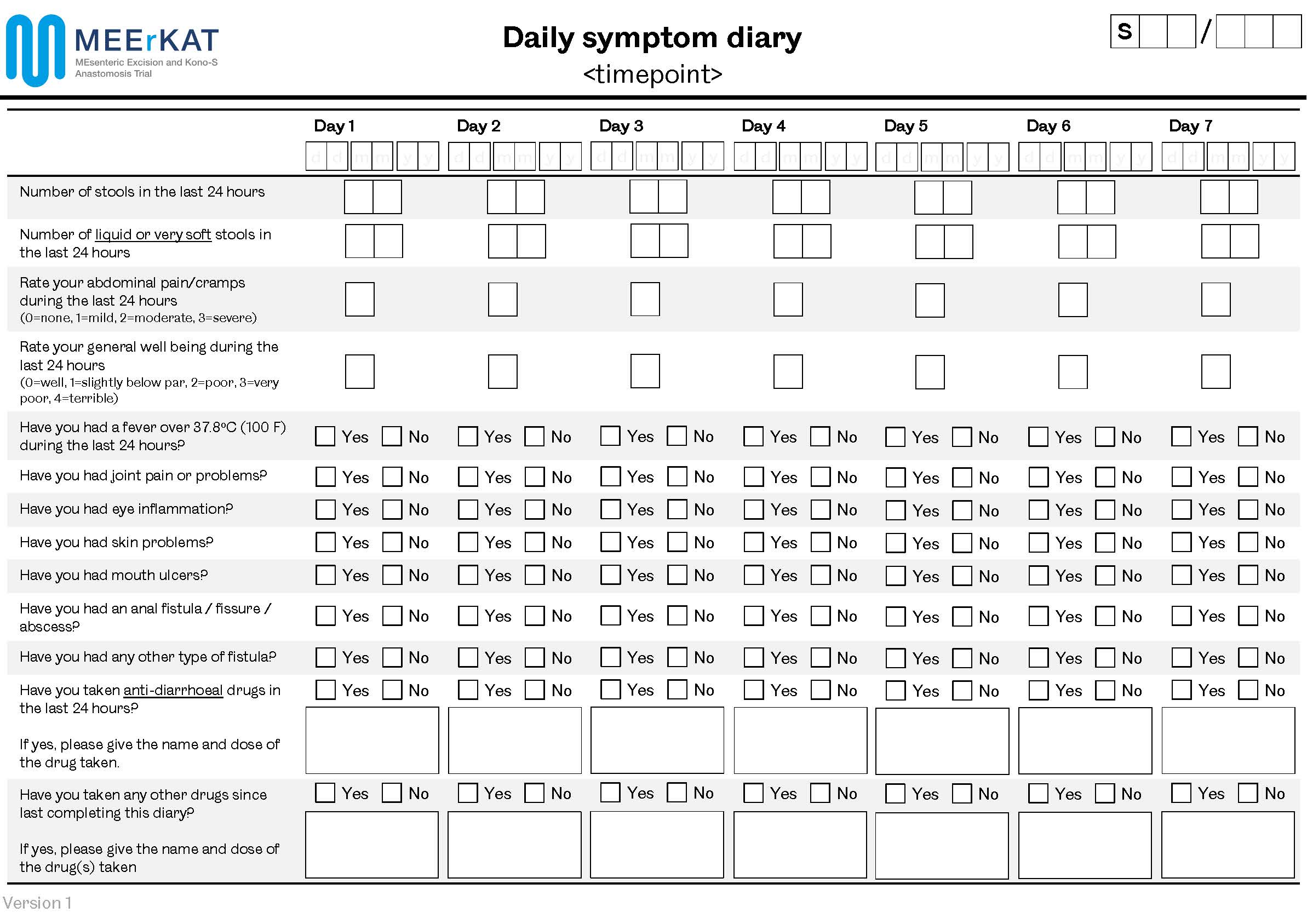
**Appendix 2. Patient symptom diary**

**Appendix 3. Collection and evaluation of biological specimens for mechanistic substudy**

All patients will undergo tattooing of the mesenteric border around the anastomosis using carbon black. Subsequent standard follow up involves an assessment for endoscopic recurrence at around 12 months [1]. This will allow localisation of any recurrence in relation to the tattoo. Endoscopic recurrence will be noted as: consistent with the hypothesis (confined to the tattooed mesenteric border only; confined to sites including the tattooed mesenteric border but more severe at the mesenteric border); refutes hypothesis (recurrence seen more than 2cm from the tattoo with the tattooed mesenteric border being recurrence free; confined to sites including the tattooed mesenteric border but less severe recurrence seen within 2cm of the tattoo); or, confined to sites including the tattooed mesenteric border of equivalent severity. To understand the mechanistic basis of intervention efficacy we will assess the sequential events between surgery and colonoscopic follow up. Visceral fat area will be assessed radiologically or at operation and correlated with recurrence (using methods described in [2,3]).

For a minimum of 140 patients, samples of mucosa will be taken at the time of operation from both the mesenteric and antimesenteric borders of the resected specimen. These will be analysed using high-parameter flow cytometry to determine the immune cell populations present at each anastomotic locality. Further samples will be taken at the follow up colonoscopy from the small bowel mesenteric and antimesenteric borders.

Visceral fat area, general immune activation, and T cell activation and exhaustion will be assessed pre-operatively and compared with post-operative recurrence to confirm the previous literature indicating high visceral fat, and lower T cell exhaustion are poor prognostic markers regardless of surgical intervention.

Changes will be assessed after each intervention to examine 3 potential scenarios:

(1) there is no change in mucosal immune activation/exhaustion after each intervention;

(2) there is a reduction in immune activation/exhaustion with one or both interventions and not in the control group suggesting the intervention(s) alter(s) the immune pathway;

(3) there are changes in locality of activated immune cells, and exhausted T cells with changes on the mesenteric border suggesting a mechanism whereby an antimesenteric anastomosis may influence outcome.

References

1. De Cruz, P., M. A. Kamm, A. L. Hamilton, K. J. Ritchie, E. O. Krejany, A. Gorelik, D. Liew, L. Prideaux, I. C. Lawrance, J. M. Andrews, P. A. Bampton, P. R. Gibson, M. Sparrow, R. W. Leong, T. H. Florin, R. B. Gearry, G. Radford-Smith, F. A. Macrae, H. Debinski, W. Selby, I. Kronborg, M. J. Johnston, R. Woods, P. R. Elliott, S. J. Bell, S. J. Brown, W. R. Connell and P. V. Desmond (2015). "Crohn's disease management after intestinal resection: a randomised trial." The Lancet 385(9976): 1406-1417.
2. Li Y, Zhu W, Gong J, et al. Visceral fat area is associated with a high risk for early postoperative recurrence in Crohn's disease.Colorectal Dis 2015;17: 225-234.
3. Probert, C. S., A. Chott, J. R. Turner,et al "Persistent clonal expansions of peripheral blood CD4+ lymphocytes in chronic inflammatory bowel disease." J Immunol **157**(7): 3183-3191.
